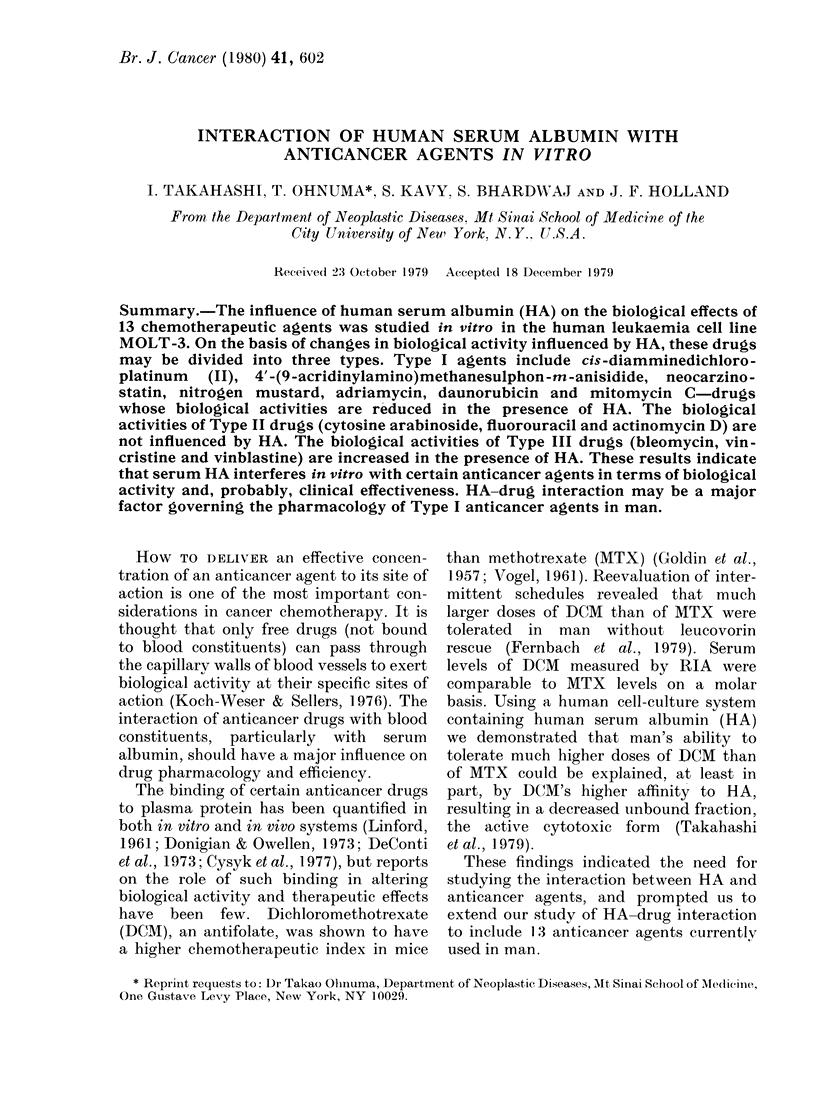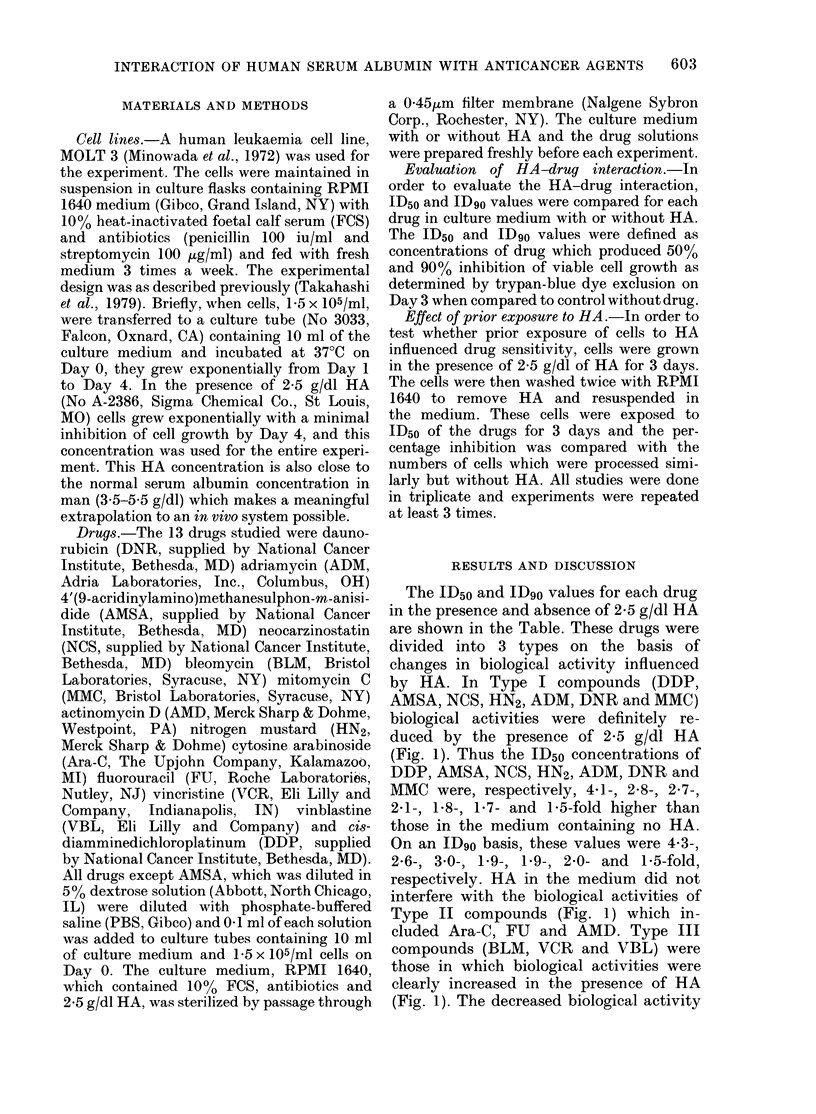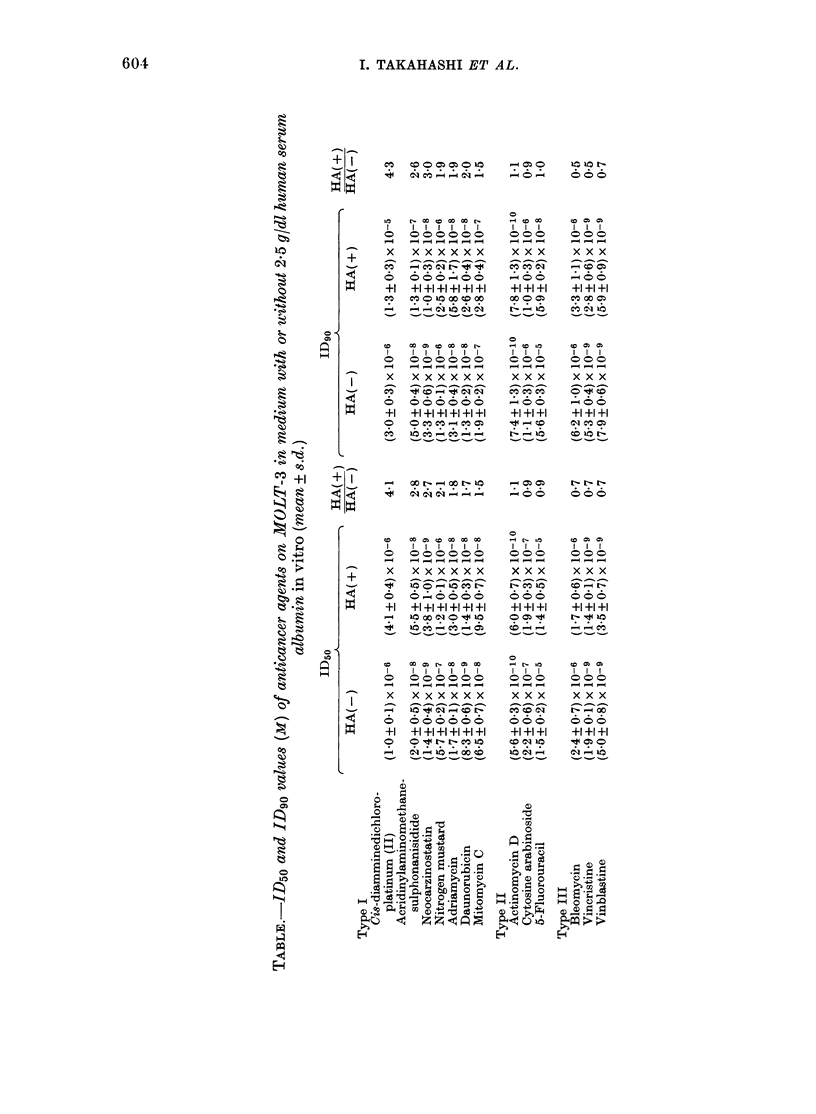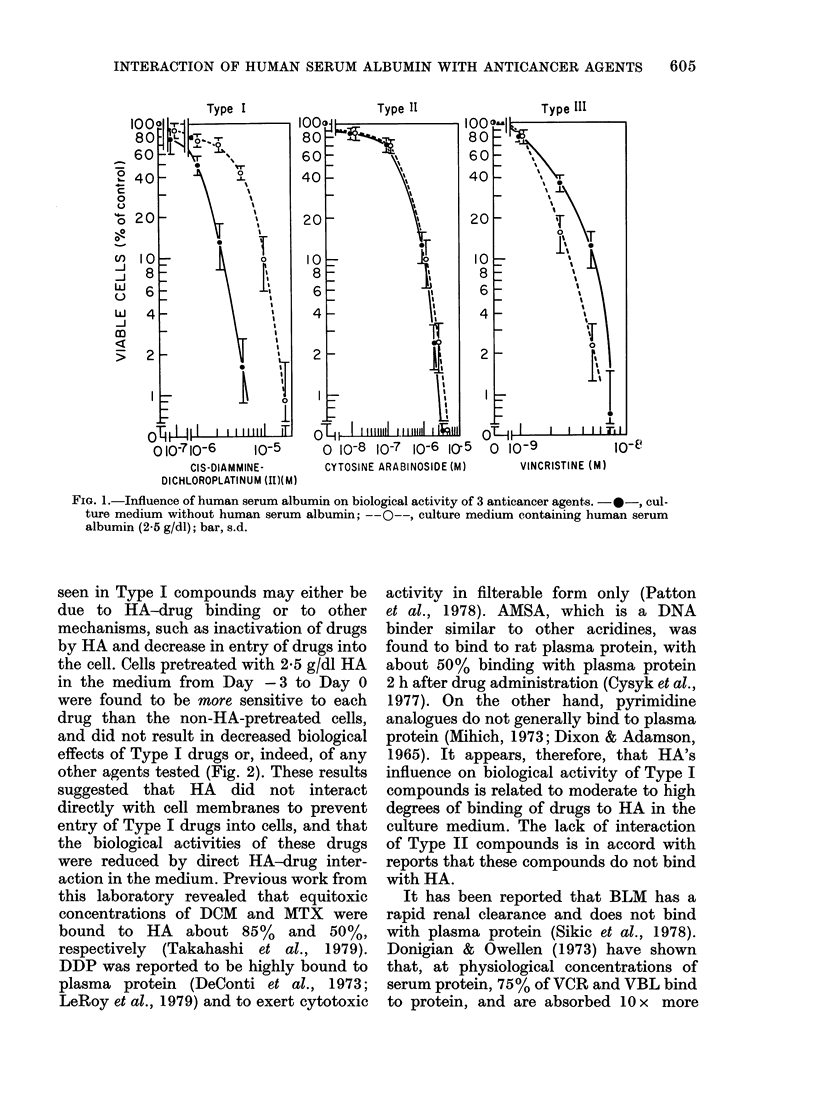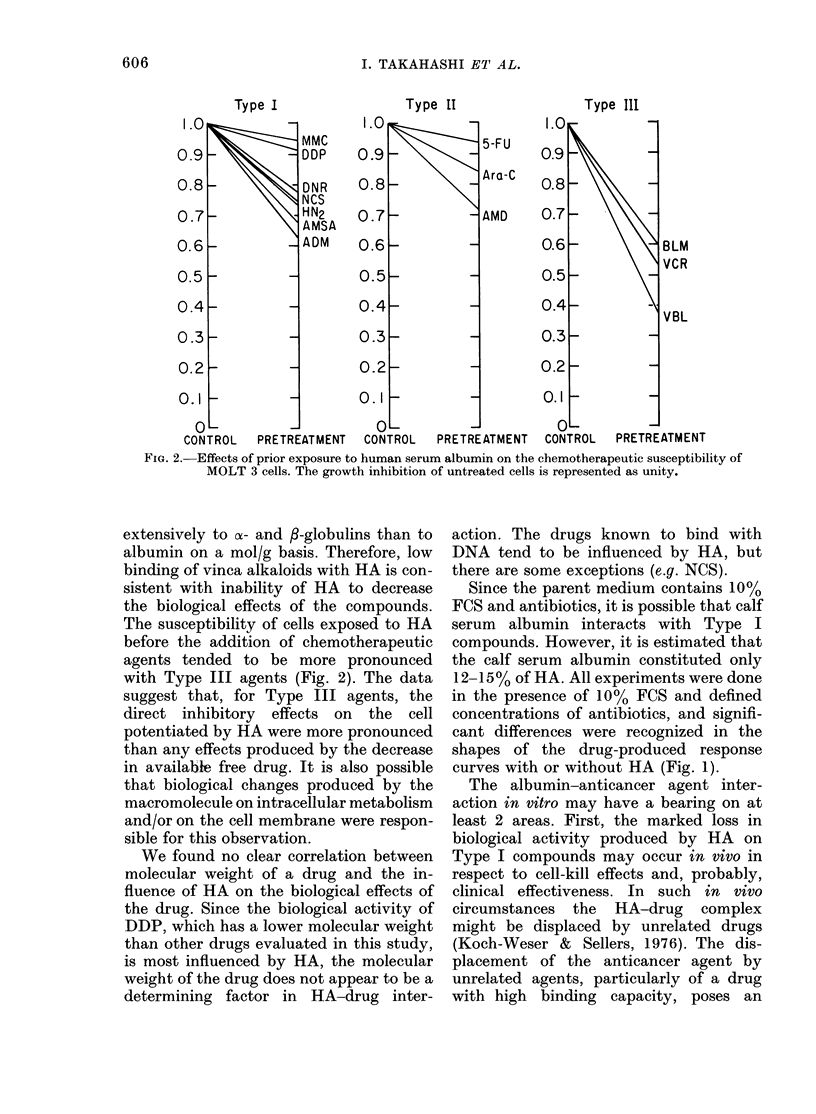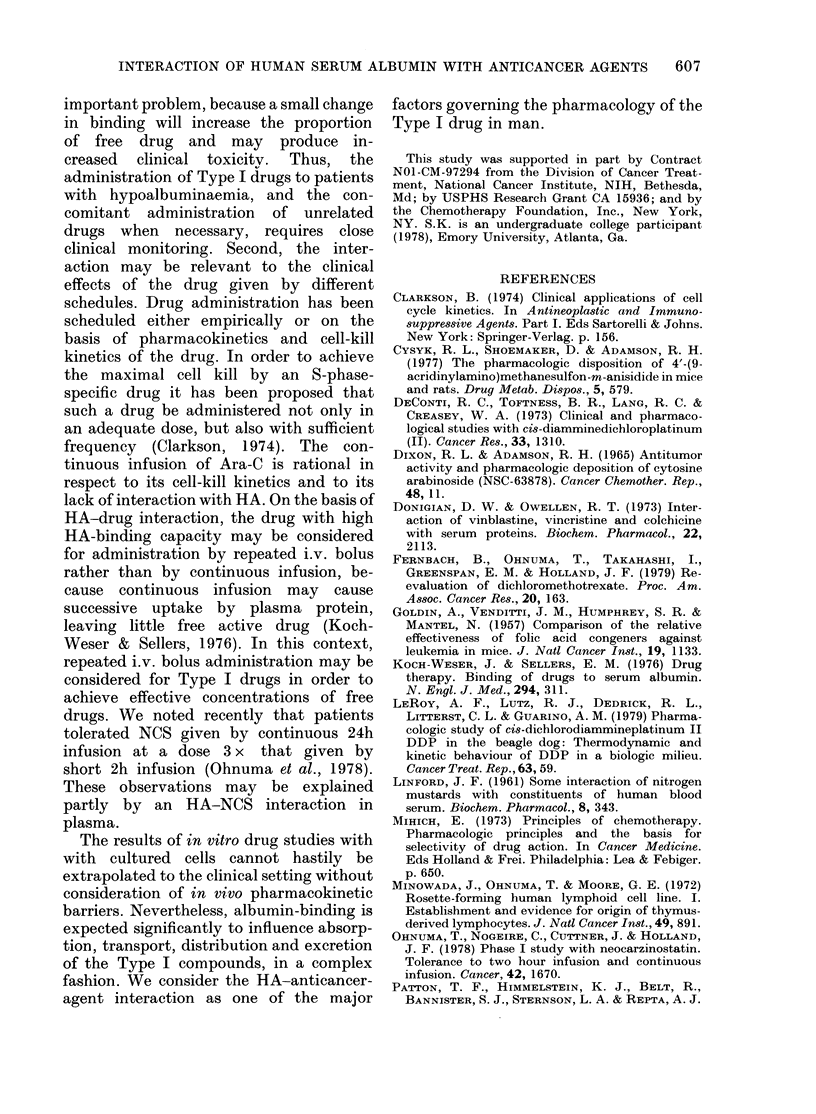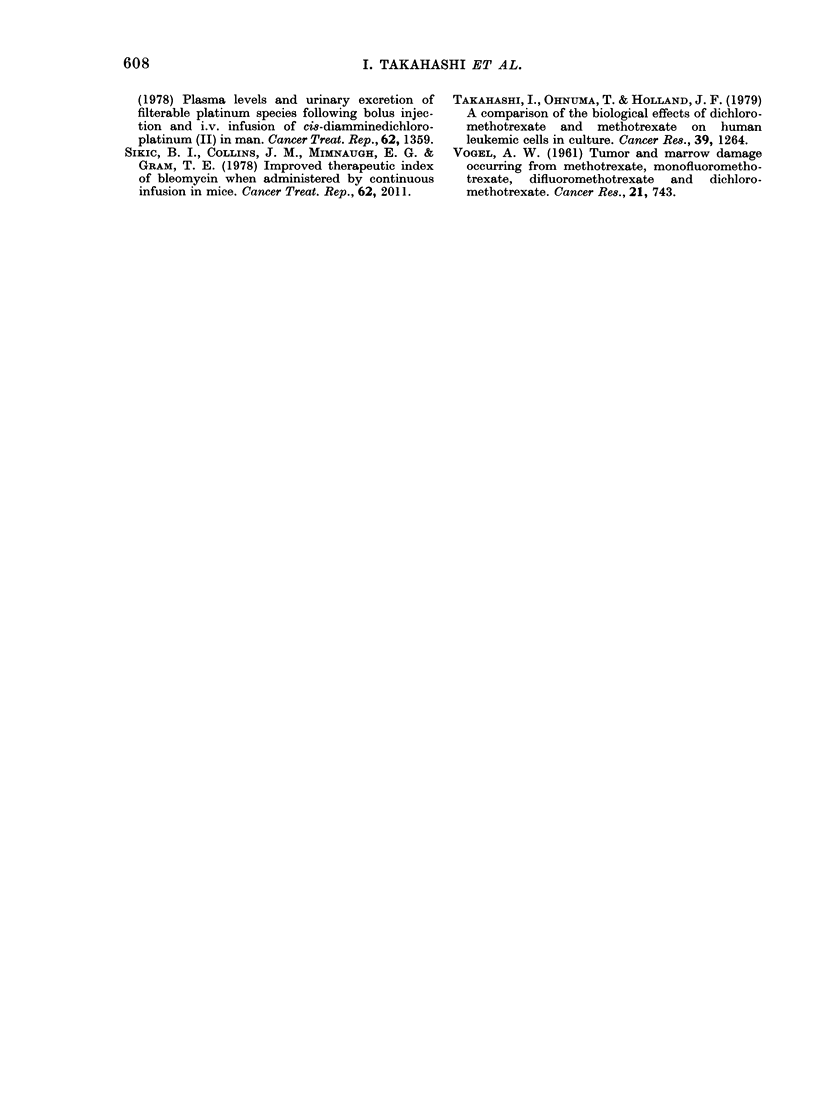# Interaction of human serum albumin with anticancer agents in vitro.

**DOI:** 10.1038/bjc.1980.103

**Published:** 1980-04

**Authors:** I. Takahashi, T. Ohnuma, S. Kavy, S. Bhardwaj, J. F. Holland

## Abstract

The influence of human serum albumin (HA) on the biological effects of 13 chemotherapeutic agents was studied in vitro in the human leukaemia cell line MOLT-3. On the basis of changes in biological activity influenced by HA, these drugs may be divided into three types. Type I agents include cis-diamminedichloroplatinum (II), 4'-(9-acridinylamino)methanesulphon-m-anisidide, neocarzinostatin, nitrogen mustard, adriamycin, daunorubicin and mitomycin C--drugs whose biological activities are reduced in the presence of HA. The biological activities of Type II drugs (cytosine arabinoside, fluorouracil and actinomycin D) are not influenced by HA. The biological activities of Type III drugs (bleomycin, vincristine and vinblastine) are increased in the presence of HA. These results indicate that serum HA interferes in vitro with certain anticancer agents in terms of biological activity and, probably, clinical effectiveness. HA-drug interaction may be a major factor governing the pharmacology of Type I anticancer agents in man.


*Br. J. Cancer* (1980) **41**, 602

### INTERACTION OF HUMAN SERUM ALBUMIN WITH ANTICANCER AGENTS *IN VITRO*

I. TAKAHASHI, T. OHNUMA*, S. KAVY, S. BHARDWAJ AND J. F. HOLLAND

*From the Department of Neoplastic Diseases, Mt Sinai School of Medicine of the City University of New York, N.Y., U.S.A.*

Received 23 October 1979 Accepted 18 December 1979

**Summary.—The influence of human serum albumin (HA) on the biological effects of 13 chemotherapeutic agents was studied *in vitro* in the human leukaemia cell line MOLT-3. On the basis of changes in biological activity influenced by HA, these drugs may be divided into three types. Type I agents include *cis*-diamminedichloroplatinum (II), 4′-(9-acridinylamino)methanesulphon-*m*-anisidide, neocarzinostatin, nitrogen mustard, adriamycin, daunorubicin and mitomycin C—drugs whose biological activities are reduced in the presence of HA. The biological activities of Type II drugs (cytosine arabinoside, fluorouracil and actinomycin D) are not influenced by HA. The biological activities of Type III drugs (bleomycin, vincristine and vinblastine) are increased in the presence of HA. These results indicate that serum HA interferes *in vitro* with certain anticancer agents in terms of biological activity and, probably, clinical effectiveness. HA–drug interaction may be a major factor governing the pharmacology of Type I anticancer agents in man.**

HOW TO DELIVER an effective concentration of an anticancer agent to its site of action is one of the most important considerations in cancer chemotherapy. It is thought that only free drugs (not bound to blood constituents) can pass through the capillary walls of blood vessels to exert biological activity at their specific sites of action (Koch-Weser & Sellers, 1976). The interaction of anticancer drugs with blood constituents, particularly with serum albumin, should have a major influence on drug pharmacology and efficiency.

The binding of certain anticancer drugs to plasma protein has been quantified in both *in vitro* and *in vivo* systems (Linford, 1961; Donigian & Owellen, 1973; DeConti *et al.*, 1973; Cysyk *et al.*, 1977), but reports on the role of such binding in altering biological activity and therapeutic effects have been few. Dichloromethotrexate (DCM), an antifolate, was shown to have a higher chemotherapeutic index in mice than methotrexate (MTX) (Goldin *et al.*, 1957; Vogel, 1961). Reevaluation of intermittent schedules revealed that much larger doses of DCM than of MTX were tolerated in man without leucovorin rescue (Fernbach *et al.*, 1979). Serum levels of DCM measured by RIA were comparable to MTX levels on a molar basis. Using a human cell-culture system containing human serum albumin (HA) we demonstrated that man's ability to tolerate much higher doses of DCM than of MTX could be explained, at least in part, by DCM's higher affinity to HA, resulting in a decreased unbound fraction, the active cytotoxic form (Takahashi *et al.*, 1979).

These findings indicated the need for studying the interaction between HA and anticancer agents, and prompted us to extend our study of HA–drug interaction to include 13 anticancer agents currently used in man.

* Reprint requests to: Dr Takao Ohnuma, Department of Neoplastic Diseases, Mt Sinai School of Medicine, One Gustave Levy Place, New York, NY 10029.

### MATERIALS AND METHODS

*Cell lines.*—A human leukaemia cell line, MOLT 3 (Minowada *et al.*, 1972) was used for the experiment. The cells were maintained in suspension in culture flasks containing RPMI 1640 medium (Gibco, Grand Island, NY) with 10% heat-inactivated foetal calf serum (FCS) and antibiotics (penicillin 100 iu/ml and streptomycin 100 $\mu$g/ml) and fed with fresh medium 3 times a week. The experimental design was as described previously (Takahashi *et al.*, 1979). Briefly, when cells, $1{\cdot}5 \times 10^5$/ml, were transferred to a culture tube (No 3033, Falcon, Oxnard, CA) containing 10 ml of the culture medium and incubated at 37°C on Day 0, they grew exponentially from Day 1 to Day 4. In the presence of 2·5 g/dl HA (No A-2386, Sigma Chemical Co., St Louis, MO) cells grew exponentially with a minimal inhibition of cell growth by Day 4, and this concentration was used for the entire experiment. This HA concentration is also close to the normal serum albumin concentration in man (3·5–5·5 g/dl) which makes a meaningful extrapolation to an *in vivo* system possible.

*Drugs.*—The 13 drugs studied were daunorubicin (DNR, supplied by National Cancer Institute, Bethesda, MD) adriamycin (ADM, Adria Laboratories, Inc., Columbus, OH) 4′(9-acridinylamino)methanesulphon-*m*-anisidide (AMSA, supplied by National Cancer Institute, Bethesda, MD) neocarzinostatin (NCS, supplied by National Cancer Institute, Bethesda, MD) bleomycin (BLM, Bristol Laboratories, Syracuse, NY) mitomycin C (MMC, Bristol Laboratories, Syracuse, NY) actinomycin D (AMD, Merck Sharp & Dohme, Westpoint, PA) nitrogen mustard ($HN_2$, Merck Sharp & Dohme) cytosine arabinoside (Ara-C, The Upjohn Company, Kalamazoo, MI) fluorouracil (FU, Roche Laboratories, Nutley, NJ) vincristine (VCR, Eli Lilly and Company, Indianapolis, IN) vinblastine (VBL, Eli Lilly and Company) and *cis*-diamminedichloroplatinum (DDP, supplied by National Cancer Institute, Bethesda, MD). All drugs except AMSA, which was diluted in 5% dextrose solution (Abbott, North Chicago, IL) were diluted with phosphate-buffered saline (PBS, Gibco) and 0·1 ml of each solution was added to culture tubes containing 10 ml of culture medium and $1{\cdot}5 \times 10^5$/ml cells on Day 0. The culture medium, RPMI 1640, which contained 10% FCS, antibiotics and 2·5 g/dl HA, was sterilized by passage through a 0·45$\mu$m filter membrane (Nalgene Sybron Corp., Rochester, NY). The culture medium with or without HA and the drug solutions were prepared freshly before each experiment.

*Evaluation of HA–drug interaction.*—In order to evaluate the HA-drug interaction, $ID_{50}$ and $ID_{90}$ values were compared for each drug in culture medium with or without HA. The $ID_{50}$ and $ID_{90}$ values were defined as concentrations of drug which produced 50% and 90% inhibition of viable cell growth as determined by trypan-blue dye exclusion on Day 3 when compared to control without drug.

*Effect of prior exposure to HA.*—In order to test whether prior exposure of cells to HA influenced drug sensitivity, cells were grown in the presence of 2·5 g/dl of HA for 3 days. The cells were then washed twice with RPMI 1640 to remove HA and resuspended in the medium. These cells were exposed to $ID_{50}$ of the drugs for 3 days and the percentage inhibition was compared with the numbers of cells which were processed similarly but without HA. All studies were done in triplicate and experiments were repeated at least 3 times.

### RESULTS AND DISCUSSION

The $ID_{50}$ and $ID_{90}$ values for each drug in the presence and absence of 2·5 g/dl HA are shown in the Table. These drugs were divided into 3 types on the basis of changes in biological activity influenced by HA. In Type I compounds (DDP, AMSA, NCS, $HN_2$, ADM, DNR and MMC) biological activities were definitely reduced by the presence of 2·5 g/dl HA (Fig. 1). Thus the $ID_{50}$ concentrations of DDP, AMSA, NCS, $HN_2$, ADM, DNR and MMC were, respectively, 4·1-, 2·8-, 2·7-, 2·1-, 1·8-, 1·7- and 1·5-fold higher than those in the medium containing no HA. On an $ID_{90}$ basis, these values were 4·3-, 2·6-, 3·0-, 1·9-, 1·9-, 2·0- and 1·5-fold, respectively. HA in the medium did not interfere with the biological activities of Type II compounds (Fig. 1) which included Ara-C, FU and AMD. Type III compounds (BLM, VCR and VBL) were those in which biological activities were clearly increased in the presence of HA (Fig. 1). The decreased biological activity

TABLE.—*$ID_{50}$ and $ID_{90}$ values* (M) *of anticancer agents on MOLT-3 in medium with or without 2·5 g/dl human serum albumin* in vitro (*mean ± s.d.*)

| | $ID_{50}$ | | | $ID_{90}$ | | |
|---|---|---|---|---|---|---|
| | HA(−) | HA(+) | HA(+)/HA(−) | HA(−) | HA(+) | HA(+)/HA(−) |
| Type I | | | | | | |
| *Cis*-diamminedichloroplatinum (II) | $(1·0 \pm 0·1) \times 10^{-6}$ | $(4·1 \pm 0·4) \times 10^{-6}$ | 4·1 | $(3·0 \pm 0·3) \times 10^{-6}$ | $(1·3 \pm 0·3) \times 10^{-5}$ | 4·3 |
| Acridinylaminomethanesulphonanisidide | $(2·0 \pm 0·5) \times 10^{-8}$ | $(5·5 \pm 0·5) \times 10^{-8}$ | 2·8 | $(5·0 \pm 0·4) \times 10^{-8}$ | $(1·3 \pm 0·1) \times 10^{-7}$ | 2·6 |
| Neocarzinostatin | $(1·4 \pm 0·4) \times 10^{-9}$ | $(3·8 \pm 1·0) \times 10^{-9}$ | 2·7 | $(3·3 \pm 0·6) \times 10^{-9}$ | $(1·0 \pm 0·3) \times 10^{-8}$ | 3·0 |
| Nitrogen mustard | $(5·7 \pm 0·2) \times 10^{-7}$ | $(1·2 \pm 0·1) \times 10^{-6}$ | 2·1 | $(1·3 \pm 0·1) \times 10^{-6}$ | $(2·5 \pm 0·2) \times 10^{-6}$ | 1·9 |
| Adriamycin | $(1·7 \pm 0·1) \times 10^{-8}$ | $(3·0 \pm 0·5) \times 10^{-8}$ | 1·8 | $(3·1 \pm 0·4) \times 10^{-8}$ | $(5·8 \pm 1·7) \times 10^{-8}$ | 1·9 |
| Daunorubicin | $(8·3 \pm 0·6) \times 10^{-9}$ | $(1·4 \pm 0·3) \times 10^{-8}$ | 1·7 | $(1·3 \pm 0·2) \times 10^{-8}$ | $(2·6 \pm 0·4) \times 10^{-8}$ | 2·0 |
| Mitomycin C | $(6·5 \pm 0·7) \times 10^{-8}$ | $(9·5 \pm 0·7) \times 10^{-8}$ | 1·5 | $(1·9 \pm 0·2) \times 10^{-7}$ | $(2·8 \pm 0·4) \times 10^{-7}$ | 1·5 |
| Type II | | | | | | |
| Actinomycin D | $(5·6 \pm 0·3) \times 10^{-10}$ | $(6·0 \pm 0·7) \times 10^{-10}$ | 1·1 | $(7·4 \pm 1·3) \times 10^{-10}$ | $(7·8 \pm 1·3) \times 10^{-10}$ | 1·1 |
| Cytosine arabinoside | $(2·2 \pm 0·6) \times 10^{-7}$ | $(1·9 \pm 0·3) \times 10^{-7}$ | 0·9 | $(1·1 \pm 0·3) \times 10^{-6}$ | $(1·0 \pm 0·3) \times 10^{-6}$ | 0·9 |
| 5-Fluorouracil | $(1·5 \pm 0·2) \times 10^{-5}$ | $(1·4 \pm 0·5) \times 10^{-5}$ | 0·9 | $(5·6 \pm 0·3) \times 10^{-5}$ | $(5·9 \pm 0·2) \times 10^{-8}$ | 1·0 |
| Type III | | | | | | |
| Bleomycin | $(2·4 \pm 0·7) \times 10^{-6}$ | $(1·7 \pm 0·6) \times 10^{-6}$ | 0·7 | $(6·2 \pm 1·0) \times 10^{-6}$ | $(3·3 \pm 1·1) \times 10^{-6}$ | 0·5 |
| Vincristine | $(1·9 \pm 0·1) \times 10^{-9}$ | $(1·4 \pm 0·1) \times 10^{-9}$ | 0·7 | $(5·3 \pm 0·4) \times 10^{-9}$ | $(2·8 \pm 0·6) \times 10^{-9}$ | 0·5 |
| Vinblastine | $(5·0 \pm 0·8) \times 10^{-9}$ | $(3·5 \pm 0·7) \times 10^{-9}$ | 0·7 | $(7·9 \pm 0·6) \times 10^{-9}$ | $(5·9 \pm 0·9) \times 10^{-9}$ | 0·7 |

FIG. 1.—Influence of human serum albumin on biological activity of 3 anticancer agents. —●—, culture medium without human serum albumin; --○--, culture medium containing human serum albumin (2·5 g/dl); bar, s.d.

seen in Type I compounds may either be due to HA–drug binding or to other mechanisms, such as inactivation of drugs by HA and decrease in entry of drugs into the cell. Cells pretreated with 2·5 g/dl HA in the medium from Day −3 to Day 0 were found to be *more* sensitive to each drug than the non-HA-pretreated cells, and did not result in decreased biological effects of Type I drugs or, indeed, of any other agents tested (Fig. 2). These results suggested that HA did not interact directly with cell membranes to prevent entry of Type I drugs into cells, and that the biological activities of these drugs were reduced by direct HA–drug interaction in the medium. Previous work from this laboratory revealed that equitoxic concentrations of DCM and MTX were bound to HA about 85% and 50%, respectively (Takahashi *et al.*, 1979). DDP was reported to be highly bound to plasma protein (DeConti *et al.*, 1973; LeRoy *et al.*, 1979) and to exert cytotoxic activity in filterable form only (Patton *et al.*, 1978). AMSA, which is a DNA binder similar to other acridines, was found to bind to rat plasma protein, with about 50% binding with plasma protein 2 h after drug administration (Cysyk *et al.*, 1977). On the other hand, pyrimidine analogues do not generally bind to plasma protein (Mihich, 1973; Dixon & Adamson, 1965). It appears, therefore, that HA's influence on biological activity of Type I compounds is related to moderate to high degrees of binding of drugs to HA in the culture medium. The lack of interaction of Type II compounds is in accord with reports that these compounds do not bind with HA.

It has been reported that BLM has a rapid renal clearance and does not bind with plasma protein (Sikic *et al.*, 1978). Donigian & Owellen (1973) have shown that, at physiological concentrations of serum protein, 75% of VCR and VBL bind to protein, and are absorbed 10× more

FIG. 2.—Effects of prior exposure to human serum albumin on the chemotherapeutic susceptibility of MOLT 3 cells. The growth inhibition of untreated cells is represented as unity.

extensively to α- and β-globulins than to albumin on a mol/g basis. Therefore, low binding of vinca alkaloids with HA is consistent with inability of HA to decrease the biological effects of the compounds. The susceptibility of cells exposed to HA before the addition of chemotherapeutic agents tended to be more pronounced with Type III agents (Fig. 2). The data suggest that, for Type III agents, the direct inhibitory effects on the cell potentiated by HA were more pronounced than any effects produced by the decrease in available free drug. It is also possible that biological changes produced by the macromolecule on intracellular metabolism and/or on the cell membrane were responsible for this observation.

We found no clear correlation between molecular weight of a drug and the influence of HA on the biological effects of the drug. Since the biological activity of DDP, which has a lower molecular weight than other drugs evaluated in this study, is most influenced by HA, the molecular weight of the drug does not appear to be a determining factor in HA–drug interaction. The drugs known to bind with DNA tend to be influenced by HA, but there are some exceptions (*e.g.* NCS).

Since the parent medium contains 10% FCS and antibiotics, it is possible that calf serum albumin interacts with Type I compounds. However, it is estimated that the calf serum albumin constituted only 12–15% of HA. All experiments were done in the presence of 10% FCS and defined concentrations of antibiotics, and significant differences were recognized in the shapes of the drug-produced response curves with or without HA (Fig. 1).

The albumin–anticancer agent interaction *in vitro* may have a bearing on at least 2 areas. First, the marked loss in biological activity produced by HA on Type I compounds may occur *in vivo* in respect to cell-kill effects and, probably, clinical effectiveness. In such *in vivo* circumstances the HA–drug complex might be displaced by unrelated drugs (Koch-Weser & Sellers, 1976). The displacement of the anticancer agent by unrelated agents, particularly of a drug with high binding capacity, poses an important problem, because a small change in binding will increase the proportion of free drug and may produce increased clinical toxicity. Thus, the administration of Type I drugs to patients with hypoalbuminaemia, and the concomitant administration of unrelated drugs when necessary, requires close clinical monitoring. Second, the interaction may be relevant to the clinical effects of the drug given by different schedules. Drug administration has been scheduled either empirically or on the basis of pharmacokinetics and cell-kill kinetics of the drug. In order to achieve the maximal cell kill by an S-phase-specific drug it has been proposed that such a drug be administered not only in an adequate dose, but also with sufficient frequency (Clarkson, 1974). The continuous infusion of Ara-C is rational in respect to its cell-kill kinetics and to its lack of interaction with HA. On the basis of HA–drug interaction, the drug with high HA-binding capacity may be considered for administration by repeated i.v. bolus rather than by continuous infusion, because continuous infusion may cause successive uptake by plasma protein, leaving little free active drug (Koch-Weser & Sellers, 1976). In this context, repeated i.v. bolus administration may be considered for Type I drugs in order to achieve effective concentrations of free drugs. We noted recently that patients tolerated NCS given by continuous 24h infusion at a dose 3× that given by short 2h infusion (Ohnuma *et al.*, 1978). These observations may be explained partly by an HA–NCS interaction in plasma.

The results of *in vitro* drug studies with with cultured cells cannot hastily be extrapolated to the clinical setting without consideration of *in vivo* pharmacokinetic barriers. Nevertheless, albumin-binding is expected significantly to influence absorption, transport, distribution and excretion of the Type I compounds, in a complex fashion. We consider the HA–anticancer-agent interaction as one of the major factors governing the pharmacology of the Type I drug in man.

This study was supported in part by Contract N01-CM-97294 from the Division of Cancer Treatment, National Cancer Institute, NIH, Bethesda, Md; by USPHS Research Grant CA 15936; and by the Chemotherapy Foundation, Inc., New York, NY. S.K. is an undergraduate college participant (1978), Emory University, Atlanta, Ga.

### REFERENCES

Clarkson, B. (1974) Clinical applications of cell cycle kinetics. In *Antineoplastic and Immunosuppressive Agents*. Part I. Eds Sartorelli & Johns. New York: Springer-Verlag. p. 156.

Cysyk, R. L., Shoemaker, D. & Adamson, R. H. (1977) The pharmacologic disposition of 4′-(9-acridinylamino)methanesulfon-*m*-anisidide in mice and rats. *Drug Metab. Dispos.*, **5**, 579.

DeConti, R. C., Toftness, B. R., Lang, R. C. & Creasey, W. A. (1973) Clinical and pharmacological studies with *cis*-diamminedichloroplatinum (II). *Cancer Res.*, **33**, 1310.

Dixon, R. L. & Adamson, R. H. (1965) Antitumor activity and pharmacologic deposition of cytosine arabinoside (NSC-63878). *Cancer Chemother. Rep.*, **48**, 11.

Donigian, D. W. & Owellen, R. T. (1973) Interaction of vinblastine, vincristine and colchicine with serum proteins. *Biochem. Pharmacol.*, **22**, 2113.

Fernbach, B., Ohnuma, T., Takahashi, I., Greenspan, E. M. & Holland, J. F. (1979) Re-evaluation of dichloromethotrexate. *Proc. Am. Assoc. Cancer Res.*, **20**, 163.

Goldin, A., Venditti, J. M., Humphrey, S. R. & Mantel, N. (1957) Comparison of the relative effectiveness of folic acid congeners against leukemia in mice. *J. Natl Cancer Inst.*, **19**, 1133.

Koch-Weser, J. & Sellers, E. M. (1976) Drug therapy. Binding of drugs to serum albumin. *N. Engl. J. Med.*, **294**, 311.

LeRoy, A. F., Lutz, R. J., Dedrick, R. L., Litterst, C. L. & Guarino, A. M. (1979) Pharmacologic study of *cis*-dichlorodiammineplatinum II DDP in the beagle dog: Thermodynamic and kinetic behaviour of DDP in a biologic milieu. *Cancer Treat. Rep.*, **63**, 59.

Linford, J. F. (1961) Some interaction of nitrogen mustards with constituents of human blood serum. *Biochem. Pharmacol.*, **8**, 343.

Mihich, E. (1973) Principles of chemotherapy. Pharmacologic principles and the basis for selectivity of drug action. In *Cancer Medicine*. Eds Holland & Frei. Philadelphia: Lea & Febiger. p. 650.

Minowada, J., Ohnuma, T. & Moore, G. E. (1972) Rosette-forming human lymphoid cell line. I. Establishment and evidence for origin of thymus-derived lymphocytes. *J. Natl Cancer Inst.*, **49**, 891.

Ohnuma, T., Nogeire, C., Cuttner, J. & Holland, J. F. (1978) Phase I study with neocarzinostatin. Tolerance to two hour infusion and continuous infusion. *Cancer*, **42**, 1670.

Patton, T. F., Himmelstein, K. J., Belt, R., Bannister, S. J., Sternson, L. A. & Repta, A. J.

(1978) Plasma levels and urinary excretion of filterable platinum species following bolus injection and i.v. infusion of *cis*-diamminedichloroplatinum (II) in man. *Cancer Treat. Rep.*, **62,** 1359.

SIKIC, B. I., COLLINS, J. M., MIMNAUGH, E. G. & GRAM, T. E. (1978) Improved therapeutic index of bleomycin when administered by continuous infusion in mice. *Cancer Treat. Rep.*, **62,** 2011.

TAKAHASHI, I., OHNUMA, T. & HOLLAND, J. F. (1979) A comparison of the biological effects of dichloromethotrexate and methotrexate on human leukemic cells in culture. *Cancer Res.*, **39,** 1264.

VOGEL, A. W. (1961) Tumor and marrow damage occurring from methotrexate, monofluoromethotrexate, difluoromethotrexate and dichloromethotrexate. *Cancer Res.*, **21,** 743.